# Beneficial effects of once-daily liraglutide, a human glucagon-like peptide-1 analogue, on cardiovascular risk biomarkers in patients with Type 2 diabetes

**DOI:** 10.1111/j.1464-5491.2008.02484.x

**Published:** 2008-09

**Authors:** J-P Courrèges, T Vilsbøll, M Zdravkovic, T Le-Thi, T Krarup, O Schmitz, R Verhoeven, I Bugáñová, S Madsbad

**Affiliations:** * Centre Hospitalier de NarbonneNarbonne, France; † Gentofte HospitalDenmark; ‡ Novo NordiskDenmark; § Århus HospitalDenmark; ¶ Gelre ZiekenhuisApeldoorn, the Netherlands; ** Dibetologická AmbulanciaZilina, Slovakia; †† Hvidovre HospitalDenmark

Liraglutide is a once-daily, human glucagon-like peptide-1 (GLP-1) analogue. Clinical studies have demonstrated blood glucose and weight-reducing effects, improvements in pancreatic B-cell function and a low risk of hypoglycaemic events with liraglutide [1,2]. Type 2 diabetes is associated with an increased risk of cardiovascular events. Recently, studies in patients with Type 2 diabetes have shown that native GLP-1 may also have beneficial effects on the myocardium [3] and on endothelial function [4].

We present here the effect of liraglutide on biomarkers for cardiovascular risk in patients with Type 2 diabetes, as an exploratory endpoint from a broader clinical study. The design and non-cardiovascular biomarker results of this study have been described previously [1]. The trial was carried out in accordance with good clinical practice. Briefly, 165 patients with Type 2 diabetes were randomized to either placebo or 0.65 mg, 1.25 mg or 1.9 mg liraglutide for 14 weeks. Across the four treatment arms, 17–23% of the subjects were previously treated with diet and exercise and the remaining subjects with oral glucose-lowering agents. Subjects had a mean body mass index (BMI) of 28.9–31.2 kg/m^2^ and mean glycated haemoglobin (HbA_1c_) at randomization of 8.1–8.5%. The study was powered against the primary endpoint HbA_1c_, but was not powered at an 80% level for a difference of 20% for the cardiovascular biomarkers discussed here. At randomization and end of study, the following additional parameters were assessed: adiponectin, leptin, high-sensitivity C-reactive protein (hs-CRP), interleukin 6 (IL-6), tumour necrosis factor alpha (TNF-α), plasminogen activator inhibitor 1 (PAI-1) and B-type natriuretic peptide (BNP).

The data are presented in Table 1. A significant decrease in PAI-1 and BNP levels were observed following treatment with liraglutide. There was a non-significant, but dose-dependent, reduction in hs-CRP levels. There were no treatment effects on levels of adiponectin, leptin, IL-6 and TNF-α with liraglutide.

**Table 1:** Baseline levels and change from baseline level of cardiovascular risk markers after 14 weeks of treatment

	Baseline level—mean (sd)* Liraglutide vs. placebo estimates (95% CI)†*P*-value‡			
	
Cardiovascular biomarker	Placebo	0.65 mg	1.25 mg	1.90 mg
PAI-1 (U/ml)	28.2 ± 20.0	27.8 ± 32.4	22.0 ± 13.0	31.4 ± 28.4
		−14% (−35%; 14%)	−29% (−47%; −6%)	−25% (−43%; −1%)
		0.29	0.018§	0.045§
BNP (ng/l)	42.8 ± 42.7	35.1 ± 22.9	45.0 ± 38.4	40.6 ± 38.6
		−26% (−48%; 6%)	−30% (−52%; −0%)	−38% (−57%; −12%)
		0.099	0.048§	0.0085§
Adiponectin (ng/ml)	4964 ± (3877)	4895 ± 2504	5713 ± 3415	3872 ± 2210
		6% (−7%; 20%)	6% (−7%; 20%)	3% (−10%; 17%)
		0.37	0.39	0.67
Leptin (pg/ml)	16 242 ± (12 717)	11 815 ± 11 991	13 724 ± 9105.8	10 812 ± 9249.7
		14% (−2%; 32%)	26% (9%; 46%)	9% (−6%; 26%)
		0.085	0.0017§	0.27
hs-CRP (mg/l)	4.3 ± (4.0)	3.1 ± 2.1	3.9 ± 3.3	4.2 ± 4.2
		−3% (−32%; 38%)	−12% (−38%; 25%)	−20% (−44%; 14%)
		0.85	0.46	0.22
IL-6 (pg/ml)	3.6 ± (3.9)	2.5 ± 1.7	10.6 ± 52.4	2.8 ± 1.7
		−3% (−25%; 26%)	4% (−20%; 34%)	−2% (−25%; 27%)
		0.83	0.79	0.87
TNF-α (pg/ml)	2.3 ± (1.5)	2.4 ± 1.5	2.0 ± 0.9	2.4 ± 2.3
		−6% (−19%; 8%)	1% (−12%; 16%)	−4% (−17%; 11%)
		0.37	0.90	0.58

* Mean ± sd of baseline values.

† Difference from placebo in change from baseline level in per cent, with 95% confidence interval.

‡ *P*-value for change. The cardiovascular risk biomarker parameters have been log transformed before applying the statistical model. Estimates are presented as per cent change. The estimates are obtained from an anovawith treatment and previous treatment as fixed effect and baseline value as covariate.

§ Statistical significance at the 5% significance level. BNP, B-type natriuretic peptide; CI, confidence interval; hs-CRP, high-sensitivity C-reactive protein; IL-6, interleukin 6; PAI-1, plasminogen activator inhibitor 1; sd, standard deviation; TNF-α, tumour necrosis factor alpha.

This study was part of a larger clinical trial, which showed significantly improved glycaemic control and a reduction in body weight in subjects treated with liraglutide [1]. In addition, systolic blood pressure (reduction of 8 mmHg at 1.90 mg/day vs. placebo) and plasma triglycerides (reduction of 22% at 1.90 mg/day vs. placebo) were significantly reduced [1]. PAI-1 and hs-CRP are inflammatory biomarkers that are associated with an increased risk of cardiovascular disease [5]. Elevated PAI-1 levels may suppress the fibrinolytic process and thereby be associated with the development of atherosclerosis. BNP is a marker of left ventricular dysfunction and elevated levels are risk markers for cardiovascular diseases, in particular for heart failure [6]. The findings suggest that liraglutide, when used to regulate blood glucose levels in patients with Type 2 diabetes, improves certain biomarkers associated with increased cardiovascular risk. Large prospective trials are needed to confirm these results and to assess whether these effects translate into improvements in cardiovascular risk in patients with Type 2 diabetes.

## Competing interests

MZ and TL-T are employed by and hold stocks in Novo Nordisk A/S. TK is a member of advisory boards for Eli Lilly and Merck. TV has been reimbursed by Novo Nordisk and MSD for attending symposia, and for speaking, and is a member of advisory boards for MSD and Novartis. SM has served as a consultant or advisor to: Novartis Pharmaceuticals, Novo Nordisk, Merck-Sharp and Dome, Pfizer A/S, Abbott Laboratories, Sanofi-Aventis, Astra-Zeneca and Johnson & Johnson.
